# Comprehensive profiling of pre-infection antibodies identifies HIV targets associated with viremic control and viral load

**DOI:** 10.3389/fimmu.2023.1178520

**Published:** 2023-09-06

**Authors:** Wendy Grant-McAuley, William Morgenlander, Sarah E. Hudelson, Manjusha Thakar, Estelle Piwowar-Manning, William Clarke, Autumn Breaud, Joel Blankson, Ethan Wilson, Helen Ayles, Peter Bock, Ayana Moore, Barry Kosloff, Kwame Shanaube, Sue-Ann Meehan, Anneen van Deventer, Sarah Fidler, Richard Hayes, Ingo Ruczinski, Kai Kammers, Oliver Laeyendecker, H. Benjamin Larman, Susan H. Eshleman

**Affiliations:** ^1^ Department of Pathology, Johns Hopkins University School of Medicine, Baltimore, MD, United States; ^2^ Institute for Cell Engineering, Johns Hopkins University School of Medicine, Baltimore, MD, United States; ^3^ Department of Medicine, Johns Hopkins University School of Medicine, Baltimore, MD, United States; ^4^ Statistical Center for HIV/AIDS Research and Prevention, Fred Hutchinson Cancer Research Center, Seattle, WA, United States; ^5^ Zambart, University of Zambia School of Public Health, Lusaka, Zambia; ^6^ Clinical Research Department, London School of Hygiene and Tropical Medicine, London, United Kingdom; ^7^ Desmond Tutu TB Center, Department of Paediatrics and Child Health, Stellenbosch University, Western Cape, South Africa; ^8^ FHI 360, Durham, NC, United States; ^9^ Department of Infectious Disease, Imperial College London, London, United Kingdom; ^10^ Department of Infectious Disease Epidemiology, London School of Hygiene and Tropical Medicine, London, United Kingdom; ^11^ Department of Biostatistics, Johns Hopkins Bloomberg School of Public Health, Baltimore, MD, United States; ^12^ Quantitative Sciences Division, Department of Oncology, Sidney Kimmel Comprehensive Cancer Center, Johns Hopkins University School of Medicine, Baltimore, MD, United States; ^13^ Laboratory of Immunoregulation, National Institute of Allergy and Infectious Diseases, National Institutes of Health, Baltimore, MD, United States

**Keywords:** HIV, controllers, viral load, antibodies, pre-infection

## Abstract

**Background:**

High HIV viral load (VL) is associated with increased transmission risk and faster disease progression. HIV controllers achieve viral suppression without antiretroviral (ARV) treatment. We evaluated viremic control in a community-randomized trial with >48,000 participants.

**Methods:**

A massively multiplexed antibody profiling system, VirScan, was used to quantify pre- and post-infection antibody reactivity to HIV peptides in 664 samples from 429 participants (13 controllers, 135 viremic non-controllers, 64 other non-controllers, 217 uninfected persons). Controllers had VLs <2,000 copies/mL with no ARV drugs detected at the first HIV-positive visit and one year later. Viremic non-controllers had VLs 2,000 copies/mL with no ARV drugs detected at the first HIV-positive visit. Other non-controllers had either ARV drugs detected at the first HIV-positive visit (n=47) or VLs <2,000 copies/mL with no ARV drugs detected at only one HIV-positive visit (n=17).

**Results:**

We identified pre-infection HIV antibody reactivities that correlated with post-infection VL. Pre-infection reactivity to an epitope in the HR2 domain of gp41 was associated with controller status and lower VL. Pre-infection reactivity to an epitope in the C2 domain of gp120 was associated with non-controller status and higher VL. Different patterns of antibody reactivity were observed over time for these two epitopes.

**Conclusion:**

These studies suggest that pre-infection HIV antibodies are associated with controller status and modulation of HIV VL. These findings may inform research on antibody-based interventions for HIV treatment.

## Graphical Abstract

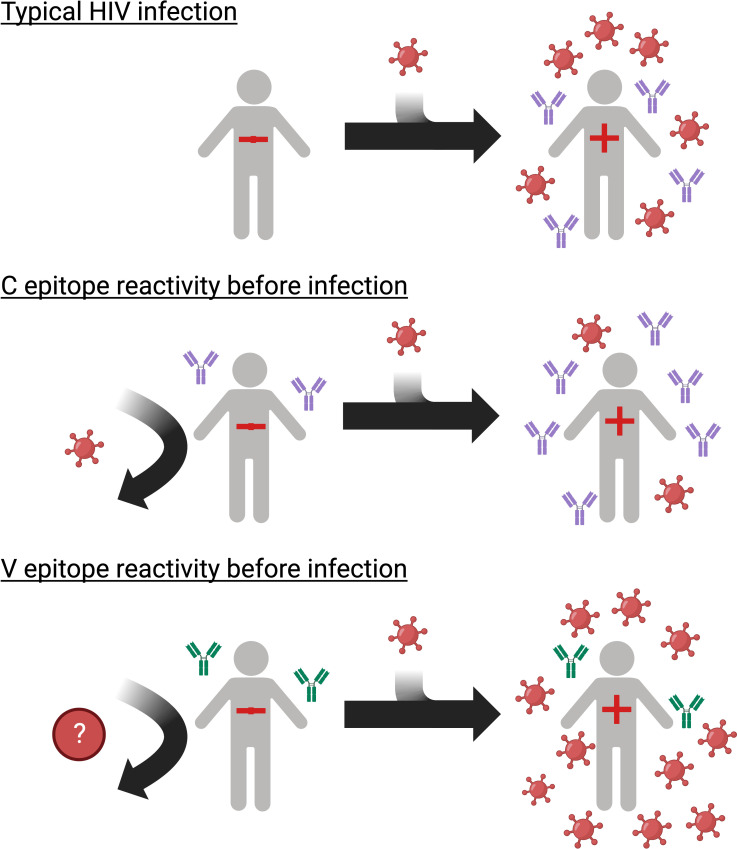

## Introduction

High HIV viral load (VL) is associated with increased transmission risk and faster disease progression. HIV controllers have low VLs without antiretroviral treatment (ART; elite controllers: <50 copies/mL; viremic controllers: <2,000 copies/mL) (1–3). HIV control develops early in infection (3, 4) and is associated with improved health outcomes (3, 5). Understanding the factors that lead to HIV control may inform the design of therapeutic HIV vaccines and antibody-based treatment strategies.

Many HIV controllers are infected with replication-competent viruses (6, 7) suggesting that host factors play a role in HIV control. Control is associated with class I HLA-B*57 and HLA-B*27 alleles (8), suggesting a role for CD8+ T cell responses. CD8+ T cell depletion in non-human primate models of elite control leads to viral rebound (9–11). Controllers also have more effective HIV-specific CD8+ T cell responses than non-controllers (12, 13) with increased cytotoxicity toward infected cells (13–15). Humoral immunity was initially not thought to play a major role in HIV control because early research described controllers with low titers of HIV-specific antibodies (16) and neutralizing antibodies (nAb) (2, 17–19), consistent with reduced antigen exposure. However, subsequent studies demonstrated higher levels of antibody dependent cellular cytotoxicity (ADCC) (20) and potent broadly neutralizing antibody (bnAb) responses (21, 22) in a subset of controllers. Antibody isotype diversity and polyfunctionality are also associated with control (23, 24). Using a massively multiplexed antibody profiling system (VirScan), we previously identified seven primary antibody targets in HIV controllers with established infection (25).

HIV-specific antibody responses develop in some persistently uninfected persons after viral exposure. In female sex workers, these antibodies exhibit cross-clade viral neutralization activity (26–28) and may (29) or may not (30) be associated with resistance to subsequent infection. In men who have sex with men, nAbs can develop following repeated oral HIV exposure (31, 32). Pre-existing ADCC in exposed infants is associated with lower rates of HIV acquisition (33, 34) and reduced morbidity after infection (33). The role of pre-infection antibodies in HIV control has not been explored.

Logistical barriers have hampered efforts to define the role of pre-existing HIV exposure-induced or cross-reactive immunity in HIV control. HIV control is typically identified in persons with established infection (3, 6, 8). Most studies include small numbers of controllers and lack pre-infection samples from controllers. A very large longitudinal study is required to identify a cohort of seroconverters who meet the criteria for HIV control and have pre-infection samples available for analysis.

The HPTN 071 (PopART) trial enrolled >48,000 participants in Zambia and South Africa (35). Participants were followed for up to three years with annual HIV testing and sample storage; 978 seroconverters were identified in the enrolled cohort (36). In this report, we used VirScan to compare pre-infection antibody reactivity in HIV controllers and viremic non-controllers in HPTN 071.

## Methods

### Source of samples

This study used samples and data from HPTN 071 (PopART) (NCT 019000977) (35). The trial was conducted in 21 urban communities in Zambia and South Africa (35), where HIV subtype C is predominant (37). The trial enrolled a population cohort of >48,000 adults (ages 18-44), randomly selected from each community, and demonstrated that population-level delivery of a combination HIV prevention package that included universal HIV testing, linkage to care and immediate offer of ART, was associated with reduced HIV incidence (35). Plasma samples were collected at annual visits from enrolled participants. VirScan was used to analyze antibody profiles in a subset of the 978 seroconverters identified in this trial. Antibody profiles were also characterized for a subset of participants who were uninfected at all study visits; participants in this group reported 0-1 lifetime sexual partners and were matched to the seroconverter group based on gender, age, and study community.

### Laboratory methods

Laboratory testing was performed at the HPTN Laboratory Center (Johns Hopkins University, Baltimore, MD). HIV status was determined at each study visit (36). Pre-infection samples additionally had no RNA detected using an assay with a limit of detection (LOD) of 400 copies/mL (36); the same assay was used to measure VL in samples collected at HIV-positive visits. VL values for HIV-positive samples with no RNA detected or RNA detected below the LOD were set at 399 copies/mL. Samples were analyzed for the presence of ARV drugs using a qualitative assay that detects 22 ARV drugs in five classes (LOD: 2 ng/mL or 20 ng/mL, depending on the drug) (38).

Samples were also analyzed using VirScan (39, 40). VirScan is a multiplexed phage-display assay that provides quantitative data for antibody binding to displayed peptides (56 amino acids long with 28 amino acid overlaps) (39, 40). The VirScan library includes peptides that span the genomes of >200 viruses that infect humans, including >3,300 peptides from multiple HIV subtypes and strains (39, 40). After sample incubation with the phage library, antibody-bound phage were immunoprecipitated using magnetic beads coated with protein A and protein G. Peptide-encoding DNA present in immunoprecipitated phage was amplified by polymerase chain reaction (PCR) using primers with sample-specific barcodes. PCR products were sequenced using the NovaSeq 6000 instrument with the S2 flowcell (Illumina, San Diego, CA) to determine the amino acid sequences of peptides bound by antibodies in study samples.

### VirScan data analysis

Longitudinal samples for each participant were included on the same 96-well immunoprecipitation plate. Each plate included 7-8 mock reactions (beads only; negative controls) and a positive control sample in triplicate (pooled plasma from 10 HPTN 071 participants with infection duration >2 years and VL >2,000 copies/mL). Antibody reactivity data for individual peptides were reported as raw read counts, fold change values (compared to read counts in observed mock reactions), and associated p-values. Raw read counts for each peptide were determined via exact matching of the first 50 nucleotides of peptide coding DNA, with one added read count for each peptide. Fold changes and p-values were determined using the exact test for the negative binomial distribution implemented in the edgeR package (41, 42). Fold change values were set at one under the following conditions: read count <15, fold change <3, and/or p-value >0.001. After adjustment, fold change values >1 indicated significant antibody binding. Z-statistics were calculated by transforming p-values.

Viral aggregate reactivity scores (VARscores) are a measure of the overall level and breadth of antibody reactivity across a viral genome. In this study, VARscores and associated p-values were calculated by comparing mean log_2_ fold change values for virus specific peptides to distributions of expected mean log_2_ fold change values for randomly selected peptides in VirScan (43). Viruses that had a VARscore greater than an empirically defined cutoff and a p-value below a Bonferroni-corrected cutoff were considered significant targets of an antibody response. Scores were calculated iteratively; in each iteration, peptides from all viruses in genera with a significantly targeted virus were removed from the pool of peptides used to generate random distributions. This process was performed a maximum of 10 iterations or until no new viruses met the reactivity thresholds. When comparing VARscores for participants with reactivity to specific peptides, those peptides were excluded from the calculation. HIV-1 VARscores were calculated as the mean VARscore across all HIV-1 subtypes.

### Statistical methods

Statistical analysis of peptide-level antibody reactivity between participant groups was performed using t-tests. Observed p-values were used to calculate multiple comparison corrected q-values to control the false discovery rate; q-values <5% were considered statistically significant (44). Alternatively, peptides of interest were selected from a cluster of peptides with observed differential reactivity (mean z-statistic for group A >1, mean z-statistic for group B <1, mean z-statistic for group A >2x that for group B). Likely antibody epitopes represented by overlapping peptides with differential reactivity were identified with epitopefindr v1.1.30 (45). Epitope reactivity was determined by selecting the maximum reactivity to any peptide with the epitope. Epitope visualization on the env trimer structure (PDB ID: 6VRW) (46) was performed with PyMOL v2.0 (47); epitope sequences from UniProt ID P04583 were aligned to the 6VRW sequence to define epitope position on the structure. VL and fold change values were log_10_-transformed prior to statistical analysis. Univariate analyses were performed using Fisher’s exact test for categorical variables or the t-test for continuous variables; paired t-tests were used for longitudinal analysis of continuous variables. Correlation analyses were performed using Pearson’s method. Statistical analyses were performed using R v4.1.2 (48). Data were visualized using ggplot2 v3.3.6 (49).

### Study approval

HPTN 071 (PopART) study participants provided written informed consent prior to enrollment. The study was approved by the institutional review boards and ethics committees at the London School of Hygiene and Tropical Medicine, the University of Zambia, and Stellenbosch University.

## Results

### Study cohort

**Figure 1:**
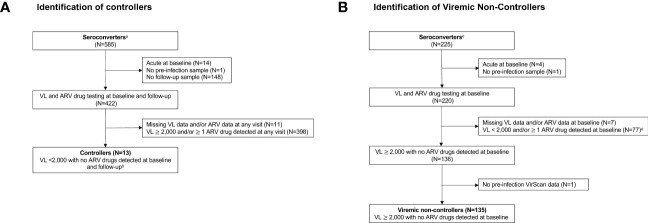
Identification of **(A)** controllers and **(B)** viremic non-controllers. Study visits were conducted during annual surveys (referred to as PC0, PC12, PC24, and PC36). The pre-infection visit was the last HIV-negative visit. The baseline visit was the first HIV-positive visit (infection duration: 0-1 year). The follow-up visit was the second HIV-positive visit (infection duration: 1-2 years). Viral load and antiretroviral drug testing were used to identify HIV controllers and viremic non-controllers. Footnotes:^a^ The 585 seroconverters included two groups (1): 360 participants were HIV negative at PC0 and HIV positive at PC12 (2); 225 participants were HIV negative at PC12 and HIV positive at PC24. The remaining 393 of 978 HPTN 071 seroconverters had different seroconversion timing and/or visit completeness and were not included. ^b^ 182 participants had an additional visit at PC36. To be classified as a controller, these participants were also required to have a VL<2,000 copies/mL with no ARV drugs detected at that visit. ^c^ The 225 seroconverters include those who were HIV negative at PC12 and HIV positive at PC24. The remaining 753 of 978 HPTN 071 seroconverters with different seroconversion timing and/or visit completeness and were not included. ^d^ Of these participants, 47 persons who were suppressed on ART at baseline (VL <400 copies/mL) and 17 non-controllers with naturally low VLs at baseline only (<2,000 copies/mL) were included in the analyses described in **Supplemental Files 7**, **8**. Abbreviations: ARV, antiretroviral; N, number; VL, viral load.

Previous analyses identified 978 seroconverters in the HPTN 071 trial (36). The approach used to identify participants for inclusion in this report is shown in **Figure 1**. Viral load and ARV drug testing were used to determine controller status for a subset of seroconverters (n=585) who were selected based on seroconversion timing and availability of samples required for analysis. Participants were classified as controllers if they had a VL <2,000 copies/mL with no ARV drugs detected at the first HIV-positive visit (baseline; infection duration 0-1 years) and the second HIV-positive visit (follow-up; infection duration 1-2 years). This approach for classifying controllers is consistent with criteria used in prior studies (2, 8, 50). Participants were classified as viremic non-controllers if they had a VL 2,000 copies/mL with no ARV drugs detected at baseline. The final cohort for the main analyses described in this report included 148 seroconverters, including 13 controllers and 135 viremic non-controllers. Some analyses described in the **Supplemental File** included an additional 47 participants who were suppressed on ART at baseline (VL <400 copies/mL), 17 non-controllers with natural suppression at baseline only (VL <2,000 copies/mL, no ARV drugs detected), and a matched control group of 217 participants who remained uninfected throughout the study.

### Association between pre-infection antibody reactivity and HIV controller status

**Figure 2:**
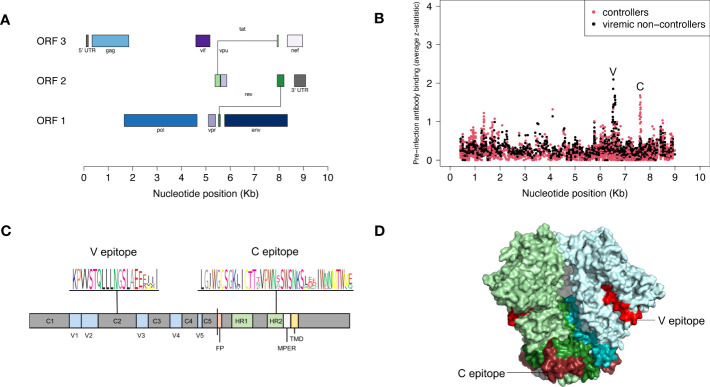
Pre-infection antibody reactivity for HIV controllers and viremic non-controllers. **(A)** The figure shows the size and position of open reading frames in the HIV genome. **(B)** The plot shows the level of pre-infection antibody binding (average z-statistic) for HIV peptides spanning the viral genome. The x-axis shows the nucleotide position in the HIV genome relative to genomic coordinates for HXB2 reference strain (NCBI #NC_001802). Each dot represents a single peptide in the VirScan library. Red dots indicate aggregate data for 13 controllers; black dots indicate aggregate data for 135 viremic non-controllers. Nine peptides had statistically significant higher antibody reactivity for viremic non-controllers; these peptides shared the V epitope (see **Supplemental File 2**). Twelve peptides had higher antibody reactivity for controllers; these peptides shared the C epitope. **(C)** The figure shows the location of the V and C epitopes in a linear model of the HIV-1 envelope protein; a consensus sequence (sequence logo) is shown for each epitope. The V epitope is in the second constant region (C2) of gp120; the C epitope is in the C-terminal heptad repeat region (HR2) of gp41. **(D)** The figure shows the location of the V and C epitopes on a cryo–electron microscopy (cryo-EM) structure of the HIV-1 envelope protein trimer (side view, PDB ID: 6VRW) (46). gp120 monomers are colored light green, light blue, and gray; gp41 monomers are colored dark green, dark blue, and gray. The V epitope is shown on all monomers in red; the C epitope is shown on all monomers in dark red. Abbreviations: C1-5, constant regions; FP, fusion protein; HR1-2, heptad repeat regions; MPER, membrane-proximal external region; ORF, open reading frame; Kb, kilobase; TMD, transmembrane domain; V1-5, variable regions.

**Figure 3:**
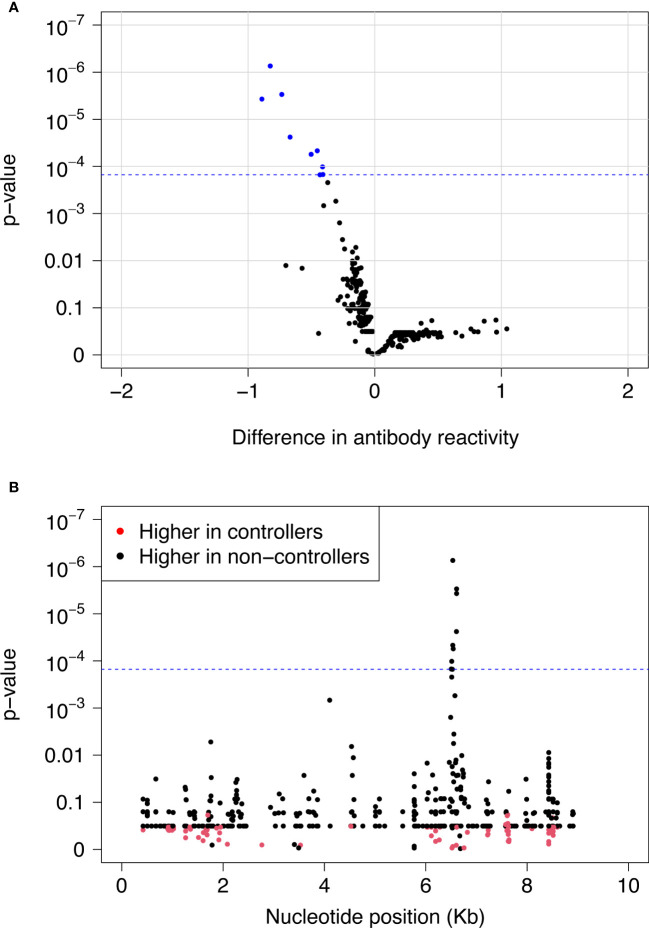
Differences in pre-infection antibody reactivity for controllers vs. viremic non-controllers. Pre-infection antibody reactivity was compared for 13 HIV controllers and 135 viremic non-controllers. **(A)** The volcano plot shows the difference in pre-infection antibody reactivity (mean fold change) between the two groups (x-axis) and the -log_10_ p-value for each peptide based on t-statistics (y-axis). Positive numbers on the x-axis correspond to stronger antibody reactivity in controllers; negative numbers on the x-axis correspond to stronger antibody reactivity in viremic non-controllers. Blue dots indicate the nine peptides that had significantly higher reactivity in viremic non-controllers compared to controllers at a false discovery rate of 5%. The blue dashed line indicates the highest q-value <5% (q=0.0418); this corresponds to a p-value of 0.000151. **(B)** The plot shows the significance of the difference in pre-infection antibody reactivity for each peptide in the VirScan library for controllers vs. viremic non-controllers. The x-axis shows the position in the HIV genome for each peptide; each dot represents a single peptide. The y-axis shows the -log_10_ p-value based on t-statistics for each peptide. Red dots indicate peptides that had higher antibody reactivity in controllers. Black dots indicate peptides that had higher antibody reactivity in viremic non-controllers. The blue dashed line indicates the highest q-value <5% (q=0.0418); this corresponds to a p-value of 0.000151. The nine peptides with q-values <0.05 (above the blue dashed line) had significantly higher reactivity in viremic non-controllers compared to controllers. These peptides contained the V epitope. Abbreviations: Kb, kilobase.

We used VirScan to compare pre-infection antibody reactivity for the 13 controllers and 135 viremic non-controllers. Both groups had low-level antibody binding to HIV peptides across the genome (**Figures 2A, B**). Two clusters of peptides were identified that had differential reactivity in these two participant groups. One cluster of nine overlapping peptides had higher levels of antibody reactivity in viremic non-controllers (V cluster); each of the individual peptides in this cluster had significantly higher mean antibody reactivity in the viremic non-controller group (q<0.05; p ≤ 0.000151; **Figure 3**). A second cluster of 12 overlapping peptides had higher mean levels of antibody reactivity in controllers (C cluster). The individual peptides in this cluster did not have significantly different antibody binding in the two groups after multiple testing correction, but had higher antibody reactivity in the controller group when the analysis was based on observed differences in z-statistics; this likely reflected the small number of controllers included in the analysis who had reactivity to these peptides.

The peptides in each cluster contained a common epitope (**Figures 2C, D**; **Supplemental File 2**). Peptides in the C cluster shared an epitope in the C-terminal heptad repeat region (HR2) of gp41 (C epitope). Peptides in the V cluster shared an epitope in the second constant (C2) region of gp120 (V epitope). Pre-infection reactivity to the C epitope (adjusted fold change >1) was observed more frequently among controllers vs. viremic non-controllers (3/13 [23.1%] vs. 5/135 [3.7%], p=0.023). Pre-infection reactivity to the V epitope was not observed among controllers but was common among viremic non-controllers (0/13 [0.0%] vs. 43/135 [31.9%], p=0.011). We did not find any sequences with homology to the C or V epitopes using BLAST, aside from HIV-1 and simian immunodeficiency virus (SIV) sequences (51).

### Association between broad pre-infection antibody reactivity to HIV peptides and HIV controller status

We next compared the depth and breadth of antibody reactivity to HIV peptides across the viral genome in the 13 controllers and 135 viremic non-controllers. This analysis was performed using an aggregate measure of antibody reactivity (VARscore); higher VARscores (generally >1) indicate prior viral exposure (52). Mean HIV-1 VARscores were low in both groups (controllers: 0.24, viremic non-controllers: 0.18, p=0.533; **Supplemental File 3**), consistent with their HIV-uninfected status.

### Association between broad pre-infection antibody reactivity to HIV and C or V epitope reactivity

**Figure 4:**
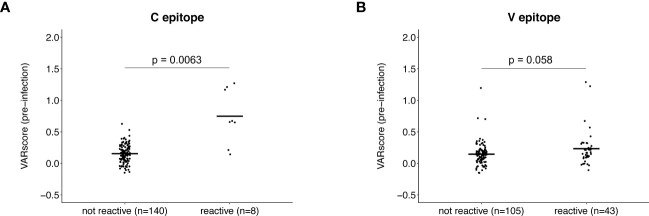
Association between antibody reactivity to the C and V epitopes and HIV-1 VARscore prior to infection. VARscores are an aggregate measure of the level and breadth of antibody reactivity to all peptides spanning the viral genome. The plots show HIV-1 VARscores prior to infection for participants with and without reactivity to the C epitope **(A)** and V epitope **(B)**. Data are shown for 148 participants (13 controllers and 135 viremic non-controllers). P-values show the significance of the association between pre-infection reactivity and HIV-1 VARscore. VARscores were calculated after excluding reactivity data for peptides containing the C (Panel A) and V epitope (Panel B).

We next compared pre-infection HIV-1 VARscores for participants with vs. without pre-infection reactivity to the C or V epitope (adjusted fold change >1 vs. adjusted fold change = 1; **Figure 4**). For this analysis, HIV-1 VARscores were calculated after removing VirScan data for peptides with the relevant epitope (C or V). First, we compared VARscores for participants with (n=8) vs. without (n=140) pre-infection reactivity to the C epitope. Participants with pre-infection reactivity to the C epitope had higher mean HIV-1 VARscores (reactive: 0.75, not reactive: 0.15, p=0.0063); this suggests that reactivity to the C epitope may have been induced by prior HIV exposure. Next, we compared VARscores for participants with (n=43) vs. without (n=105) pre-infection reactivity to the V epitope. HIV-1 VARscores were low in both groups with no difference between groups (reactive: 0.23, not reactive: 0.15, p=0.058); this suggests that reactivity to the V epitope may not have been induced by prior HIV exposure.

### Changes in antibody reactivity to the C and V epitopes after HIV infection

We next compared antibody reactivity to the C and V epitopes at the pre-infection visit and the first HIV-positive visit (baseline, infection duration 0-1 year). VirScan data were available at baseline for 145/148 participants, including 7/8 participants with pre-infection reactivity to the C epitope and 42/43 participants with pre-infection reactivity to the V epitope. (**Supplemental File 4**). VirScan data were available at follow-up for 86/148 participants, including 7/8 participants with pre-infection reactivity to the C epitope and 26/43 participants with preinfection reactivity to the V epitope.

**Figure 5:**
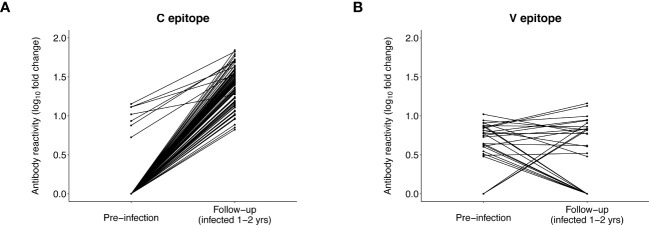
Antibody reactivity to the C and V epitopes before and after HIV infection. The plots show the level of antibody reactivity (log_10_ fold change) to the C and V epitopes at the pre-infection and follow-up visits. VirScan data were available for 86 participants at the follow-up visit (13 controllers and 73 viremic non-controllers, **Supplemental File 4**). **(A)** includes paired data for 86 participants who had reactivity to the C epitope at the pre-infection and/or follow-up visit. All 86 participants had reactivity to the C epitope at follow-up; the seven participants with reactivity to the C epitope before infection had significantly higher reactivity to this epitope at follow-up. **(B)** includes paired data for 29 participants who had reactivity to the V epitope at the pre-infection and/or follow-up visit (data are not shown for 57 participants who had no reactivity to the V epitope at either visit). Three participants who had no reactivity to the V epitope before infection developed reactivity to the V epitope at follow-up. Eleven of the 26 participants who had reactivity to the V epitope before infection no longer had reactivity to this epitope at follow-up; the remaining 15 participants did not have differential reactivity to this epitope at follow-up compared to pre-infection.

At baseline, almost all participants (143/145 [98.6%]) had C epitope reactivity; this included all seven participants with pre-infection reactivity to this epitope (**Supplemental File 5A**). Reactivity to this epitope (mean fold change) increased at baseline for the seven participants with pre-infection reactivity (pre-infection: 10.3, baseline: 29.1, p=0.0041). Mean baseline reactivity was similar for participants with vs. without pre-infection reactivity (reactive at pre-infection: 29.1, not reactive at pre-infection: 22.2, p=0.059). We then evaluated reactivity to the C epitope at follow-up (infection duration 1-2 years) when infection was more established. At follow-up, all 86 participants had reactivity to the C epitope, including all seven participants with pre-infection reactivity (**Figure 5A**). Mean reactivity was higher at follow-up than pre-infection for participants with pre-infection reactivity (pre-infection: 10.3, follow-up: 42.5, p=0.00036). Mean reactivity was also higher at follow-up for those with vs. without pre-infection reactivity (reactive at pre-infection: 42.5, not reactive at pre-infection: 25.9, p=0.0088). The findings from analysis of data from the baseline and follow-up visits are consistent with results from the VARscore analysis, indicating that HIV exposure induces and boosts expression of C epitope antibodies.

Different patterns of antibody reactivity were observed for the V epitope. Only 37/145 (25.5%) participants had reactivity to the V epitope at baseline (**Supplemental File 5B**); most (32/37 [86.5%]) of these participants also had pre-infection reactivity to this epitope. Ten additional participants with pre-infection reactivity to the V epitope were no longer reactive to this epitope at baseline. There was no increase in V epitope reactivity (mean fold change) after infection for the 32 participants who had V epitope reactivity at both visits (pre-infection: 6.4, baseline: 6.1, p=0.33). We also observed no difference in mean baseline reactivity for participants with vs. without pre-infection reactivity (reactive at pre-infection: 6.1, not reactive at pre-infection: 6.8, p=0.68). Furthermore, only 18/86 (20.9%) participants had reactivity to the V epitope at follow-up, including 15 participants with pre-infection reactivity (**Figure 5B**). An additional 11 participants with pre-infection reactivity did not have reactivity to this epitope at follow-up. Mean reactivity was unchanged at follow-up (vs. pre-infection) for the 15 participants with reactivity at both visits (pre-infection: 6.5, follow-up: 7.3, p=0.68), and there was no difference in mean follow-up reactivity for participants with vs. without pre-infection reactivity (reactive at pre-infection: 7.3, not reactive at pre-infection: 7.2, p=0.58). These findings are consistent with results from the VARscore analysis, indicating that expression of V epitope antibodies is not induced or boosted by HIV exposure or HIV infection.

### Association between antibody reactivity to the C and V epitopes and viral load

**Figure 6:**
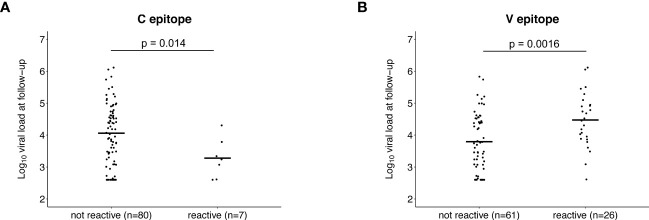
Association between pre-infection antibody reactivity to the C and V epitopes and HIV viral load. The plots show the association between the presence of antibody reactivity to the C epitope **(A)** and V epitope **(B)** before infection (pre-infection visit) and log_10_ HIV viral load after infection (follow-up visit; infection duration: 1-2 years). Data are shown for the 87 participants who did not have antiretroviral drugs detected at follow-up and had viral load data from that visit (13 controllers and 74 viremic non-controllers, **Supplemental File 4**). P-values show the significance of the association between pre-infection reactivity and HIV viral load at follow-up.

We next evaluated whether pre-infection reactivity to the C and V epitopes was associated with VL after infection. Participants with pre-infection reactivity to the C epitope had lower mean VLs at baseline than those without pre-infection reactivity, but the difference was not statistically significant (reactive at pre-infection: 23,969 copies/mL, not reactive at pre-infection: 98,982 copies/mL, p=0.16; **Supplemental File 6A**). A significant difference was observed for mean VL at follow-up (reactive at pre-infection: 4,594 copies/mL, not reactive at pre-infection: 81,124 copies/mL, p=0.014; **Figure 6A**). These findings indicate that the pre-infection reactivity to the C epitope is associated with lower VLs in established infection.

The opposite association was observed for the V epitope. Participants with pre-infection reactivity to the V epitope had higher mean VLs at baseline but the difference was not statistically significant (reactive at pre-infection: 119,243 copies/mL, not reactive at pre-infection: 84,969 copies/mL, p=0.068; **Supplemental File 6B**). A significant difference was observed for mean VL at follow-up (reactive at pre-infection: 150,647 copies/mL, not reactive at pre-infection: 42,709 copies/mL, p=0.0016; **Figure 6B**). These findings indicate that the pre-infection reactivity to the V epitope is associated with higher VLs in established infection.

**Figure 7:**
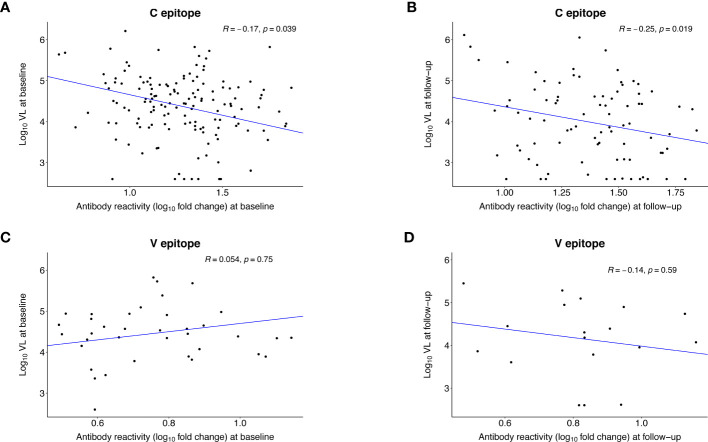
Correlation between the strength of antibody reactivity to the C and V epitopes after infection and HIV viral load. The plots show the Pearson correlation (R) between the strength of antibody reactivity (log_10_ fold change) to the C epitope **(A, B)** or the V epitope **(C, D)** and log_10_ HIV viral load. VirScan data were available for 145 (98%) of 148 participants at baseline and 86 (58%) of 148 participants at follow-up (**Supplemental File 4**). Each dot represents data for one participant; participants who did not have reactivity to the epitope of interest were excluded from the analysis. The blue line indicates the first principal component. **(A)** shows data for the 143 participants who had reactivity to the C epitope at the baseline visit (infection duration: <1 year). **(B)** shows data for the 86 participants who had reactivity to the C epitope at the follow-up visit (infection duration: 1-2 years). **(C)** shows data for the 37 participants who had reactivity to the V epitope at the baseline visit. **(D)** shows data for the 18 participants who had reactivity to the V epitope at the follow-up visit.

We also evaluated whether the strength of antibody reactivity to the C and V epitopes at baseline or follow-up was correlated with VL at the same visit (**Figure 7**). In persons with C epitope reactivity, the strength of reactivity was inversely correlated with VL at baseline (R= -0.17, p=0.039) and follow-up (R= -0.25, p=0.019). There was no observed correlation between the strength of reactivity to the V epitope and VL at either visit.

### Association between pre-infection antibody reactivity and infection risk

As a final step, we evaluated whether pre-infection antibody reactivity was associated with subsequent HIV infection (**Supplemental File 7**). There were no differences in mean antibody reactivity for 212 seroconverters vs. 217 uninfected participants to any HIV peptides in the VirScan library, including those with the C or V epitopes. Mean pre-infection HIV-1 VARscores were also low with no difference between groups (seroconverters: 0.186, uninfected: 0.217 p=0.134; **Supplemental File 8**).

## Discussion

Previous studies demonstrated that HIV-specific pre-existing antibody responses can be protective in non-controllers (26–29, 31–34) but did not include a comprehensive evaluation of reactivity to all expressed HIV peptides. In this study, we used VirScan to compare pre-infection antibody profiles in controllers and viremic non-controllers in HPTN 071. All participants, independent of controller or infection status, had low-level antibody binding to HIV peptides across the viral genome prior to infection; this may reflect a combination of HIV-specific and non-specific binding to target peptides. A subset of participants had pre-infection antibodies to the C and V epitopes; this reactivity was associated with HIV controller status and VL after infection. Neither of these epitopes is targeted by bnAbs under investigation for HIV treatment and prevention (53, 54).

We found that the C epitope (gp41, HR2) was targeted more frequently prior to infection in controllers vs. viremic non-controllers (23.1% vs. 3.7%). Pre-infection reactivity to this epitope was also associated with higher overall HIV-specific antibody responses prior to infection, suggesting that production of C epitope antibodies was stimulated by HIV exposure without infection. At follow-up (infection duration: 1-2 years), pre-infection responses were boosted and all participants had reactivity to this epitope. Pre- and post-infection reactivity to the C epitope were also associated with lower VL after infection.

The 12 peptides with the C epitope overlap with an HR2 epitope that was targeted in controllers with established HIV infection in our prior study (25). HR2 plays a critical role in viral fusion; antibodies targeting this region may impair hairpin formation and viral entry (55). NAbs targeting HR2 have been described in slow progressors (56) and controllers (57), while non-neutralizing HR2 antibodies with robust ADCC activity were found in a non-controller (58). This report demonstrates that pre-infection antibody reactivity to the C epitope is associated with controller status and lower VL after infection, consistent with antibody-mediated suppression of viral replication.

Antibodies to V epitope (gp120, C2) were only present prior to infection in viremic non-controllers. In contrast to the C epitope, pre-infection reactivity to this epitope was not associated with pre-infection reactivity to other HIV peptides, suggesting that production of V epitope antibodies was triggered by a mechanism other than HIV exposure. Changes in reactivity to the V epitope over time were also different from those observed for the C epitope. At follow-up, pre-infection responses to the V epitope were not boosted or consistently maintained, and only three participants developed V epitope reactivity after infection. Overall, these data suggest that V epitope reactivity was triggered by an unidentified, non-HIV antigen.

The nine peptides with the V epitope (C2 target) did not overlap with epitopes identified in our previous work. However, in the RV144 trial (NCT00223080; Thailand), vaccination induced protective antibodies targeting C2; these antibodies were associated with higher levels of ADCC and reduced HIV acquisition risk (59). This contrasts with the findings in our study, where pre-existing V-epitope reactivity was not protective and was instead associated with higher post-infection viral loads and non-controller status. These conflicting results may reflect subtype-specific differences in antibody responses targeting C2.

Pre-existing non-neutralizing or cross-reactive antibodies can sometimes facilitate viral entry by interacting with complement and/or the Fc receptor through antibody dependent enhancement (ADE) (60). In HIV infection, ADE allows rapid viral trafficking by antigen presenting cells (e.g., macrophages) to lymph node resident CD4+ T cells following mucosal transmission, enhancing early processes required to establish robust infection (61–64). Infection-enhancing antibodies targeting env have been associated with HIV transmission risk (65) and higher VL after infection (66). We find that pre-infection reactivity to the V epitope is associated with non-controller status and higher VL after infection. Taken together, our findings suggest that V epitope reactivity might modulate VL via ADE in early infection.

This study had several limitations. First, negative HIV status pre-infection was determined using a sensitive antigen/antibody assay and an RNA assay with a LOD of 400 copies/mL; it is possible that some participants with low RNA and antibody levels could have been misclassified as uninfected. Second, the controller cohort used for peptide discovery was very small (n=13); this may have limited our ability to identify other pre-infection antibody specificities associated with HIV control. Third, the study cohort only included participants with presumed HIV subtype C; as inter-subtype genetic diversity may influence antibody responses, further studies are needed to assess generalizability of the findings in this study to persons with other HIV subtypes. Fourth, the VirScan assay measures antibody binding to unglycosylated, linear epitopes; antibody binding detected with VirScan may not correlate with antibody binding to the native HIV envelope trimer. Fifth, the phage-display library used in this report did not include HIV antisense protein peptides (67). Sixth, most prior studies of pre-infection HIV antibodies have focused on mucosal IgA responses (26–29, 34); this report was limited to the analysis of plasma IgG reactivity and did not include other isotypes or mucosal responses. Seventh, we did not evaluate other factors known to contribute to viral load modulation (e.g., HLA haplotype (8), cellular immune responses to HIV infection (12–15), viral factors (68, 69)). Finally, we did not assess antibody profiles in viremic controllers identified in HPTN 071 who were HIV positive at study enrollment; that analysis will be described in a separate report.

## Conclusion

We identified two env epitopes that were targeted in some persons prior to infection. Reactivity to one epitope was associated with natural control of HIV infection and appeared to reduce VL via antibody-mediated suppression of viral replication. In contrast, reactivity to the other epitope was not present in HIV controllers and appeared to increase VL, possibly via ADE early in infection. These findings enhance our understanding of humoral mechanisms impacting controller status and VL and could inform the design of antibody-based approaches for HIV treatment.

## Data availability statement

The raw data supporting the conclusions of this article will be made available by the authors, without undue reservation.

## Ethics statement

The studies involving humans were approved by the institutional review boards and ethics committees at the London School of Hygiene and Tropical Medicine, the University of Zambia, and Stellenbosch University. The studies were conducted in accordance with the local legislation and institutional requirements. The participants provided their written informed consent to participate in this study.

## Author contributions

WG-M: designed the study, performed testing, analyzed data, drafted the manuscript. WM: performed testing, analyzed data. SEH: performed testing. MT: performed testing. EP-M: HPTN 071 (PopART) QAQC representative, responsible for HIV and viral load testing. WC: responsible for antiretroviral drug testing. AB: performed antiretroviral drug testing. JB: provided expertise on viremic control. EW: HPTN 071 (PopART) data analyst. HA: HPTN 071 (PopART) site PI for Zambia. PB: HPTN 071 (PopART) site PI for South Africa. AM: HPTN 071 (PopART) study manager. BK: provided laboratory support for HPTN 071 (PopART) in Zambia. KS: HPTN 071 (PopART) Zambia site investigator. S-AM: HPTN 071 (PopART) research manager. AV: provided laboratory support for HPTN 071 (PopART) in South Africa. SF: HPTN 071 (PopART) protocol co-chair. RH: HPTN 071 (PopART) protocol chair. IR: provided statistical and bioinformatics expertise, analyzed data. KK: provided statistical and bioinformatics expertise, analyzed data. OL: designed the study, provided input into data analysis. HBL: designed the study, analyzed data. SHE: designed the study, analyzed data, drafted the manuscript. All authors contributed to the manuscript and approved the submitted version.
