# Supplementary material for: Comprehensive profiling of pre-infection antibodies identifies HIV targets associated with viremic control and viral load
Source: Front Immunol. 2023 Sep 6;14:1178520. doi: 10.3389/fimmu.2023.1178520 (PMC10512082; doi:10.3389/fimmu.2023.1178520)
Supplement: Supplementary file 1 [file DataSheet_1.pdf]

Supplementary Material

Comprehensive profiling of pre-infection antibodies identifies HIV targets associated with viremic control and viral load

Wendy Grant-McAuley[1], William Morgenlander[1,2], Sarah E. Hudelson[1], Manjusha Thakar[1], Estelle Piwowar-Manning[1], William Clarke[1], Autumn Breaud[1], Joel Blankson[3], Ethan Wilson[4], Helen Ayles[5,6], Peter Bock[7], Ayana Moore[8], Barry Kosloff[5,6], Kwame Shanaube[5], Sue Ann Meehan[7], Anneen van Deventer[7], Sarah Fidler[9], Richard Hayes[10], Ingo Ruczinski[11], Kai Kammers[12], Oliver Laeyendecker[3,13], H. Benjamin Larman[1,2], Susan H. Eshleman[1*], for the HPTN 071 (PopART) Study Team

[*] **Correspondence**
Corresponding author:
Susan Eshleman, MD/PhD
seshlem@jhmi.edu

Supplemental File 1. HPTN 071 (PopART) Study Team

Name	Affiliation
Richard Hayes	London School of Hygiene and Tropical Medicine (LSHTM)
Sarah Fidler	Imperial College
Nulda Beyers	Desmond Tutu Tuberculosis Centre
Helen Ayles	ZAMBART
Peter Bock	Desmond Tutu Tuberculosis Centre
Wafaa El-Sadr	HIV Prevention Trials Network (HPTN) Leadership and Operations Center (LOC)
Myron Cohen	HPTN LOC
Susan Eshleman	HPTN Laboratory Center (LC)
Yaw Agyei	HPTN LC
Estelle Piwowar-Manning	HPTN LC
Virginia Bond	ZAMBART
Graeme Hoddinott	Desmond Tutu Tuberculosis Centre
Deborah Donnell	HPTN Statistical and Data Management Center (SDMC)
Sian Floyd	LSHTM
Ethan Wilson	HPTN SDMC
Lynda Emel	HPTN SDMC
Heather Noble	HPTN SDMC
Dave McLeod	LSHTM
David Burns	NIAID
Christophe Fraser	Oxford University
Anne Cori	Imperial College
Nirupama Deshmane Sista	HPTN LOC
Sam Griffith	HPTN LOC
Ayana Moore	HPTN LOC
Tanette Headen	HPTN LOC
Rhonda White	HPTN LOC
Eric Miller	HPTN LOC
James Hargreaves	LSHTM
Katharina Hauck	Imperial College
Ranjeeta Thomas	Imperial College
Mohammed Limbada	ZAMBART
Justin Bwalya	ZAMBART
Michael Pickles	University of Manitoba
Kalpana Sabapathy	LSHTM
Ab Schaap	ZAMBART
Rory Dunbar	Desmond Tutu Tuberculosis Centre
Kwame Shanaube	ZAMBART
Blia Yang	Desmond Tutu Tuberculosis Centre
Musonda Simwinga	ZAMBART
Peter C. Smith	Imperial College Business School
Sten Vermund	HPTN

Nomtha Mandla	Desmond Tutu Tuberculosis Centre
Nozizwe Makola	Desmond Tutu Tuberculosis Centre
Anneen van Deventer	Desmond Tutu Tuberculosis Centre
Anelet James	Desmond Tutu Tuberculosis Centre
Karen Jennings	City Health Department, City of Cape Town
James Kruger	Department of Health, Western Cape
Mwelwa Phiri	ZAMBART
Barry Kosloff	ZAMBART
Lawrence Mwenge	ZAMBART
Sarah Kanema	ZAMBART
Rafael Sauter	Oxford University
Will Probert	Oxford University
Ramya Kumar	ZAMBART
Ephraim Sakala	ZAMBART
Andrew Silumesi	Ministry of Health, Zambia
Tim Skalland	HPTN SDMC
Krista Yuhas	HPTN SDMC

Supplemental File 2. Characteristics of peptides with differential antibody reactivity in controllers vs. viremic non-controllers.

Peptide ID	HIV gene location	HIV protein location	Amino acid position	HXB2 coordinates	Amino acid sequence	UniProt Number	Epitope
77383	env	gp120; C2	225-280	6419-6587	YCAPAGFAILKCNNNTCNGTGPCTNVSTVQCTHGIKPVVSTQLLLNGSLAEEEIII	Q9QBZ8	V
23202	env	gp120; C2	225-280	6428-6596	PAGFAILKCNDKKFNGTEICKNVSTVQCTHGIKPVVSTQLLLNGSLAEEEIMIRSE	P04583	V
77292	env	gp120; C2	225-280	6440-6608	AILKCNNKTFNGTGLCRNVSTVQCTHGIKPVVSTQLLLNGSLAEEKMIIRSENISD	Q9QBZ0	V
17461	env	gp120; C2	225-280	6449-6617	KCNNKTFNGTGPCNNVSTIQCTHGTKPVVSTQLLLNGSLAEEEIIIRSKNLTDNVK	O12164	V
18298	env	gp120; C2	225-280	6449-6617	KCRDKEYNGTGPCKNVSTVQCTHGIKPVVSTQLLLNGSLAEEDIRIRSENFTDNTK	O41803	V
31665	env	gp120; C2	225-280	6458-6626	DKKFNGTGPCTNVSTVQCTHGIKPVVSTQLLLNGSLAEGEVVIRSENFTNNAKTII	P12490	V
77239	env	gp120; C2	253-308	6521-6689	IKPVVSTQLLLNGSLAEEEIIIRSENITDNTKNIIVQLNETVQINCTRPNNNTRKS	Q9QBY2	V
35887	env	gp120; C2	253-308	6524-6692	KPVVSTQLLLNGSLAEEEVVIRSDNFTNNAKTILVQLNVSVEINCTRPNNNRRRRI	P19549	V
77293	env	gp120; C2	253-308	6524-6692	KPVVSTQLLLNGSLAEEKMIIRSENISDNTKTIIVQFKNPVKINCTRPNNNTRRSI	Q9QBZ0	V
77250	env	gp41; HR2	561-616	7493-7661	QLRARILAVERYLKDQQLLGIWGCSGKLICTTNVPWNSSWSNKSWEEIWNNMTWME	Q9QBY2	C
78242	env	gp41; HR2	561-616	7523-7691	RYLKDQQLLGIWGCSGKLICTTNVPWNSSWSNKSQEEIWNNMTWMEWEKEISNYSN	Q9QSQ7	C
23125	env	gp41; HR2	589-644	7508-7676	VLAVERYLRDQQLLGIWGCSGKLICTTTVPWNASWSNKSLNMIWNNMTWMQWEREI	P04579	C
24026	env	gp41; HR2	589-644	7514-7682	AVESYLKDQQLLGIWGCSGKHICTTTVPWNSSWSNKSLEEIWNNMTWIEWEREIDN	P05882	C
22110	env	gp41; HR2	589-644	7520-7688	ERYLKDQQLLGIWGCSGKLICTTAVPWNASWSNKSLEQIWNNMTWMEWDREINNYT	P03377	C
76908	env	gp41; HR2	589-644	7526-7694	YLKDQQLLGIWGCSGKLICTTNVPWNSSWSNKSLDEIWDNMTWMEWDKQINNYTDE	Q9Q714	C
23215	env	gp41; HR2	589-644	7529-7697	LQDQRLLGMWGCSGKHICTTFVPWNSSWSNRSLDDIWNNMTWMQWEKEISNYTGII	P04583	C
23936	env	gp41; HR2	589-644	7532-7700	KDQQLLGFWGCSGKLICTTTVPWNASWSNKSLDDIWNNMTWMQWEREIDNYTSLIY	P05877	C
23996	env	gp41; HR2	589-644	7535-7703	DQQLLGIWGCSGKIICPTNVPWNSSWSNKSQSDIWDKMTWLEWDKEVSNYTQVIYN	P05881	C
41895	env	gp41; HR2	589-644	7535-7703	DQQLLGIWGCSGKLICTTTVPWNASWSNKSMDQIWNNMTWMEWEREIDNYTSLIYN	P31872	C
22140	env	gp41; HR2	589-644	7538-7706	QQLLGIWGCSGKLICTTAVPWNASWSNKSLEDIWDNMTWMQWEREIDNYTNTIYTL	P03378	C
35899	env	gp41; HR2	589-644	7547-7715	LGIWGCSGKLICTTTVPWNTSWSNKSLDKIWNNMTWMEWEREIDNYTSLIYTLLEE	P19549	C

Table Legend for Supplemental File 2.

The table shows the characteristics of the 21 peptides that had different levels of antibody reactivity in controllers vs. viremic non-controllers. Twelve peptides had higher reactivity in controllers (peptides with the C epitope). Nine peptides had significantly higher reactivity in viremic non-controllers (peptides with the V epitope). The following information is provided for each peptide: Peptide identifier; HIV gene location; HIV protein location; amino acid position; HXB2 coordinates (HXB2, NCBI #NC_001802); amino acid sequence; UniProt number; epitope designation (C or V). V and C epitope sequences are shown in red font.

Abbreviations: ID: identifier; env: HIV-1 envelope protein; gp120: glycoprotein 120; C2: second constant region, gp41: glycoprotein 41; HR2: C-terminal heptad repeat region.

Supplemental File 3. Association between controller status and HIV-1 VARscore prior to infection.

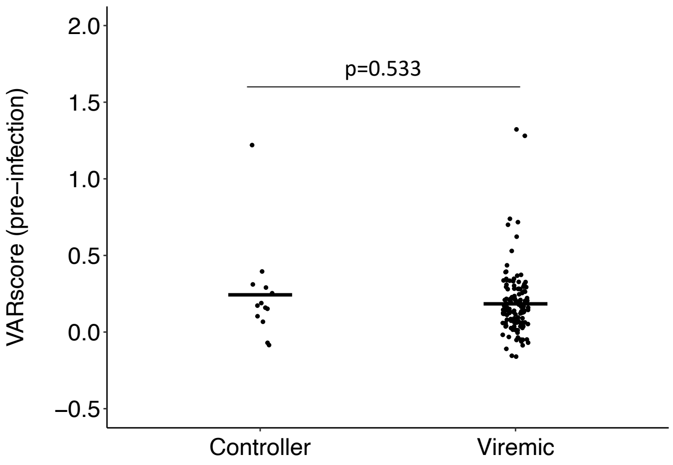

Legend for Supplemental File 3:

VARscores are an aggregate measure of the level and breadth of antibody reactivity to all peptides spanning the viral genome. The plots show HIV-1 VARscores prior to infection for participants classified as controllers (n=13) and viremic non-controllers (n=135). P-values show the significance of the association between controller status and HIV-1 VARscore.

Supplemental File 4. Available data by study group and visit.

Study visit	Assay	Controllers (N=13)	Viremic non-controllers (N=135)
Pre-infection	VirScan	13	135
Baseline	VirScan	13	132[a]
	Viral load	13	135
Follow-up	VirScan	13	73[b]
	Viral load	13	74[c]

Table Legend for Supplemental File 4:
The table shows the number of participants with data from VirScan and viral load testing by HIV controller status and study visit.

Footnotes:

[a] Three viremic non-controllers were not included in the analysis of VirScan data at baseline due to assay failure. This included one participant with pre-infection reactivity to the C epitope and one participant with pre-infection reactivity to the V epitope.

[b] Sixty-two viremic non-controllers were not included in the analysis of VirScan data at follow-up for the following reasons: no follow-up visit (N=27), on antiretroviral therapy (N=34), assay failure (N=1). This included one participant with pre-infection reactivity to the C epitope and 17 participants with pre-infection reactivity to the V epitope.

[c] Sixty-one viremic non-controllers were not included in the analysis of viral load at follow-up for the following reasons: no follow-up visit (N=27), on antiretroviral therapy (N=34). This included one participant with pre-infection reactivity to the C epitope and 17 participants with pre-infection reactivity to the V epitope.

Supplemental File 5. Antibody reactivity to the C and V epitopes before and after HIV infection (baseline visit).

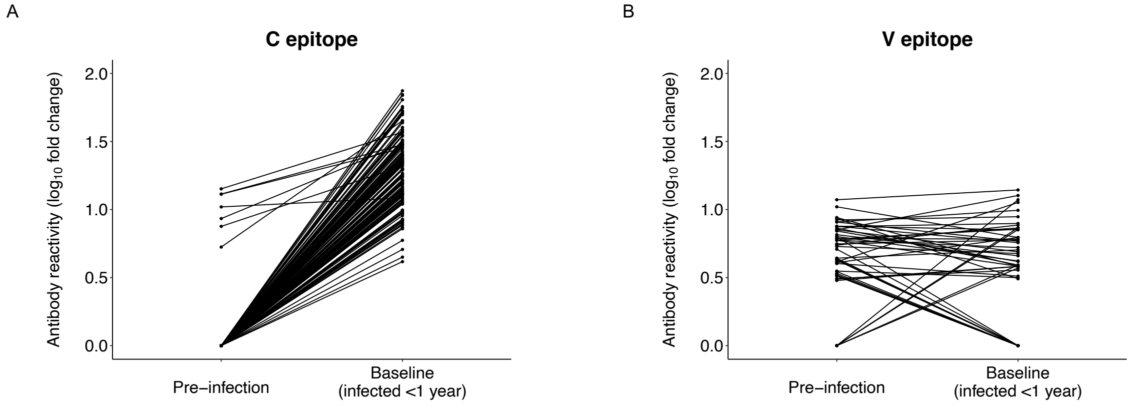

Figure Legend for Supplemental File 5:
The plots show the level of antibody reactivity ($\log_{10}$ fold change) to the C and V epitopes at the pre-infection and baseline visits. VirScan data were available for 145 participants at the baseline visit (13 controllers and 132 viremic non-controllers, Supplemental File 4).
Panel A includes paired data for 143 participants who had reactivity to the C epitope at the pre-infection and/or baseline visit (data are not shown for the 2 participants who had no reactivity to the C epitope at either visit). The seven participants with reactivity to the C epitope at pre-infection did not have differential reactivity to this epitope at baseline compared to pre-infection.
Panel B includes paired data for 37 participants who had reactivity to the V epitope at the pre-infection and/or baseline visit (data are not shown for 108 participants who had no reactivity to the V epitope at either visit). Five participants who had no reactivity to the V epitope before infection developed reactivity to the V epitope at baseline. Ten of the 42 participants who had reactivity to the V epitope before infection no longer had reactivity to this epitope at baseline; the remaining 15 participants did not have differential reactivity to this epitope at baseline compared to pre-infection.

Supplemental File 6. Association between pre-infection antibody reactivity to the C and V epitopes and HIV viral load (baseline visit).

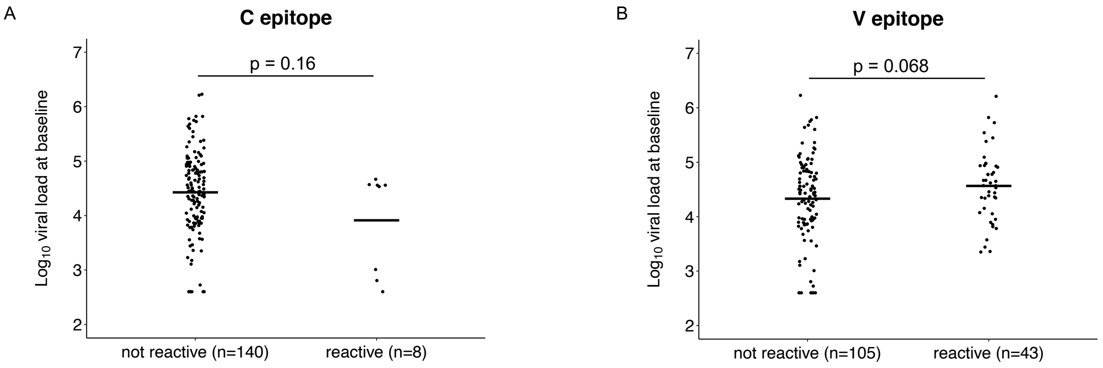

Figure Legend for Supplemental File 6:

The plots show the association between the presence of antibody reactivity to the C epitope (Panel A) and V epitope (Panel B) before infection (pre-infection visit) and $\log_{10}$ HIV viral load after infection (baseline visit; infection duration: <1 year). Data are shown for 145 participants (13 controllers, 132 viremic non-controllers). P-values show the significance of the association between pre-infection reactivity and HIV viral load at baseline.

Supplemental File 7. Pre-infection antibody reactivity for seroconverters and participants who did not acquire HIV infection during the study.

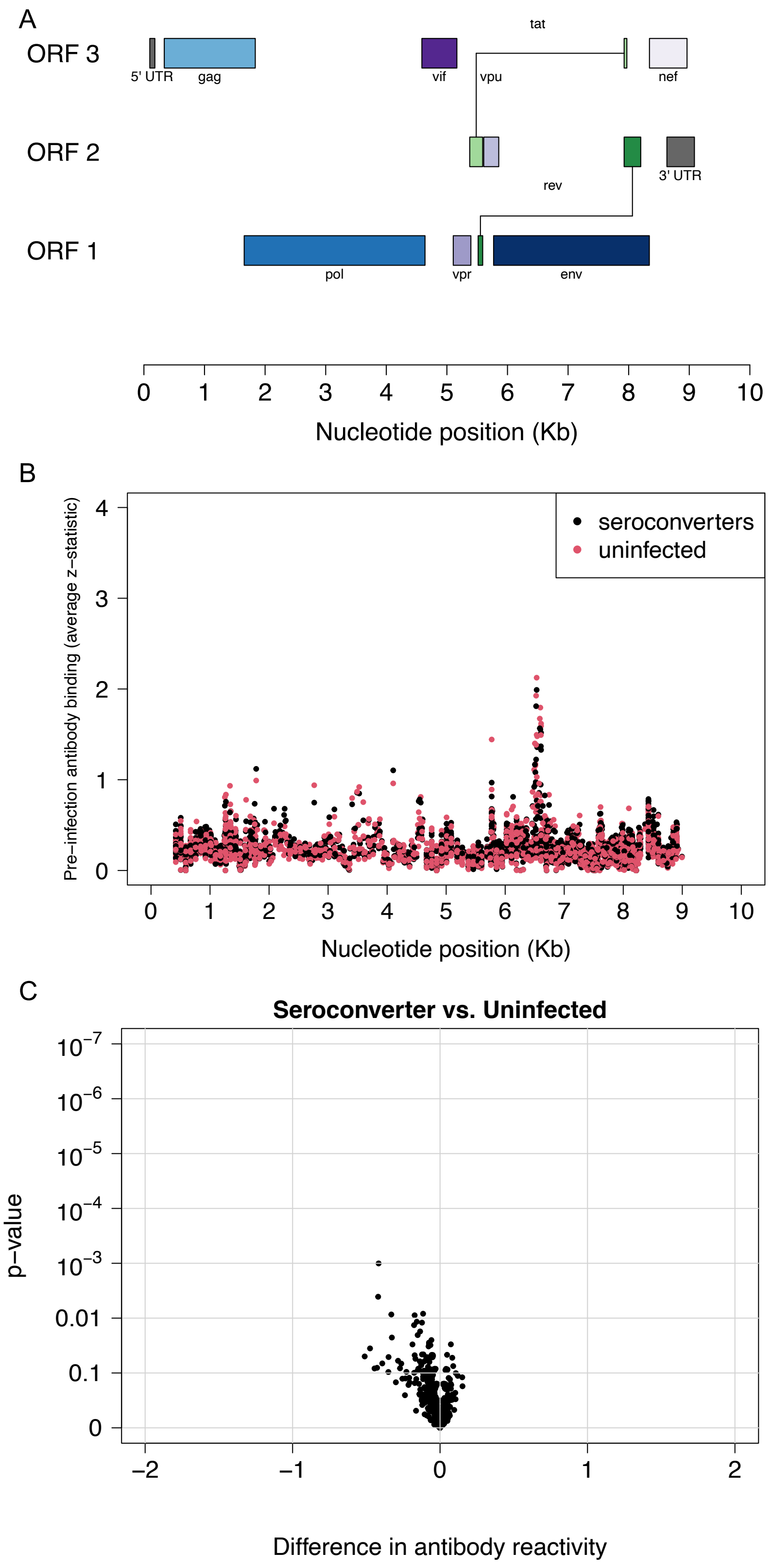

Figure legend for Supplemental File 7.

We evaluated the association between pre-infection antibody reactivity and infection risk. This analysis included an expanded group of participants who acquired HIV infection during HPTN 071 (13 controllers and 135 viremic non-controllers included in the main analyses, plus 64 additional non-controllers who either had ARV drugs detected at baseline [n=47] or were naturally suppressed at baseline only [n=17]; total 212 seroconverters). This analysis also included 217 participants who were uninfected at all study visits; participants in the uninfected group reported 0-1 lifetime sexual partners and were matched to the 212 seroconverters based on gender, age, and study community. Participants in both groups had low-level antibody binding to HIV peptides across the genome prior to infection (Panel B). There were no differences in antibody reactivity to individual peptides between the 212 seroconverters and the 217 uninfected participants (Panel C).

Panel A. The figure shows the size and position of open reading frames in the HIV genome.

Panel B. The plot shows the level of pre-infection antibody binding (average z-statistic) for HIV peptides spanning the viral genome. The x-axis shows the nucleotide position in the HIV genome relative to genomic coordinates for HXB2 reference strain (NCBI #NC_001802). Each dot represents a single peptide in the VirScan library. Black dots indicate data for the 212 seroconverters (13 controllers and 199 non-controllers); red dots indicate data for the 217 uninfected participants.

Panel C. The volcano plot shows the difference in antibody reactivity (fold change) between the two groups (x-axis) and the $-\log_{10}$ p value for each peptide based on t-statistics (y-axis). Positive numbers correspond to stronger antibody reactivity in seroconverters.

Abbreviations: ORF: open reading frame; Kb: kilobase.

Supplemental File 8. Association between subsequent HIV status and HIV-1 VARscore.

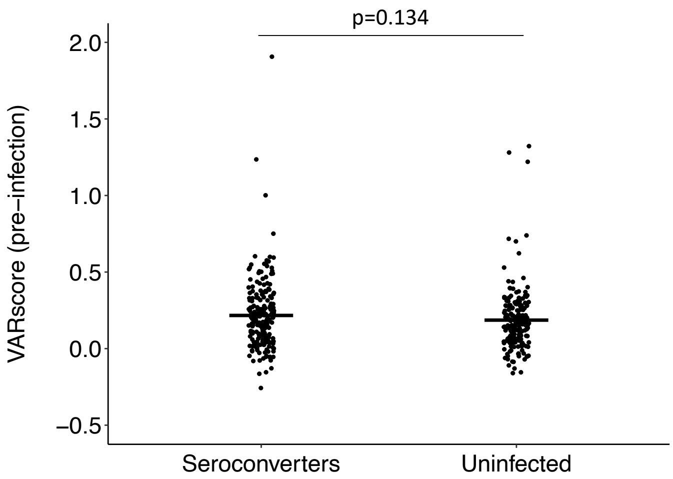

Legend for Supplemental File 8:

VARscores are an aggregate measure of the level and breadth of antibody reactivity to all peptides spanning the viral genome. The plots show HIV-1 VARscores for 212 seroconverters and 217 uninfected participants (see Supplemental File 7). This analysis was completed to explore whether HIV exposure prior to infection was associated with subsequent HIV acquisition. P-values show the significance of the association between subsequent HIV status and HIV-1 VARscore. These findings indicate that prior HIV exposure was not associated with HIV infection risk.
